# Effects of propolis-modified glass ionomer cement on antimicrobial activity and physico-mechanical properties: a systematic review

**DOI:** 10.1007/s10266-025-01209-y

**Published:** 2025-09-30

**Authors:** Dina Abozaid, Enas Elwakeel, Maged Ahmed Mohamed, Mohammad A. Bahnsawy, Mohamed Eldebawy, Alaaeldin Elraggal, Fatima Alzahraa Abdellah Gwidah, Amr Azab

**Affiliations:** 1 Dental Biomaterials Department, Faculty of Dentistry, Tanta University, Tanta, Egypt; 2 Faculty of Dentistry, Tanta University, Tanta, Egypt; 3 Faculty of Dental Medicine, Al-Azhar University, Cairo, Egypt; 4 Medical Research Group of Egypt, Negida Academy LLC, Massachusetts Avenue, Arlington, MA 02474 USA; 5 Division of Dentistry, School of Medical Sciences, Faculty of Biology, Medicine and Health, The University of Manchester, Manchester, UK; 6 Conservative Dentistry Department, Faculty of Dentistry, Alexandria University, Alexandria, Egypt; 7 Prosthodontics Department, Faculty of Dentistry, Tanta University, Tanta, Egypt

**Keywords:** Glass ionomer cement, Propolis, Antimicrobial activity, Mechanical properties, Physical properties

## Abstract

Glass ionomer cement (GIC) is valued in restorative dentistry for its chemical adhesion, fluoride release, and biocompatibility but has limitations in mechanical strength and long-term antibacterial activity. Propolis, a natural resin rich in flavonoids and phenolic acids, has shown antimicrobial, antioxidant, and anti-inflammatory effects, making it a potential bioactive additive to improve GIC performance. This systematic review evaluates the effects of propolis-modified GIC on antimicrobial activity and physico-mechanical properties, while identifying optimal formulations and concentrations for clinical use. Special emphasis is placed on methodological heterogeneity, chemical characterization gaps, and future research needs. Publications up to the end of 2024 were included from the Cochrane Library, Web of Science, PubMed, and Scopus. The review was registered on PROSPERO (CRD42024627498) and conducted according to PRISMA guidelines using the PICO framework. Propolis-modified GIC generally demonstrated enhanced antibacterial activity against *Streptococcus mutans*, *Lactobacillus*, and *Candida albicans*, particularly at concentrations of 25–50%. Mechanical outcomes varied with concentration, with 25% often balancing antimicrobial efficacy and mechanical stability, while higher concentrations improved hardness but sometimes reduced compressive and bond strength. Considerable heterogeneity was observed in GIC brands, propolis sources, extraction techniques, and incorporation methods, limiting direct comparability. Most studies lacked detailed chemical characterization, making it unclear which flavonoids or phenolic compounds drive the observed effects. Yellow discoloration was the main reported esthetic change, with no studies assessing long-term color stability or translucency. Propolis-modified GIC is a promising bioactive restorative material with potential antimicrobial benefits. However, methodological weaknesses, heterogeneity in preparation, and absence of chemical and long-term esthetic evaluation limit the direct clinical applicability of current evidence. Standardized protocols for material preparation, bioactive component identification, and comprehensive testing of mechanical and esthetic properties are recommended to advance this field.

## Introduction

Glass Ionomer Cement (GIC) has become a fundamental material in restorative dentistry, appreciated for its combination of unique properties such as fluoride release, biocompatibility, and chemical adhesion to tooth structures [1]. Structurally, GIC is considered as hybrid materials that are primarily composed of both organic and inorganic components, typically consisting of calcium fluoro-alumino-silicate glass powder combined with aqueous solutions of homo- and copolymers of acrylic acid, often including tartaric acid. While GIC does not strictly meet the definition of true ionomers from a chemical perspective, they are more accurately categorized as glass polyalkenoate cements [2]. However, the conventional designation as “GIC” remains widely used [3].

Over the past five decades, GIC has been widely used in numerous dental applications, including as liners, bases, luting agents, sealers, and restorative materials [4]. Their popularity stems from their strong adhesion to tooth structure, antimicrobial properties through fluoride release, thermal compatibility with dental tissues, and overall biocompatibility [5]. Despite these advantages, GIC exhibits several drawbacks, such as susceptibility to moisture, limited strength, long-term wear, and intrinsic opacity [5]. Moreover, while GIC’s self-curing properties and fluoride release offer potential for caries inhibition, concerns remain about its effectiveness in completely preventing recurrent caries [6, 7]. Studies have shown that residual bacteria can remain viable beneath GIC restorations for a maximum of two years, highlighting the persistent risk of secondary caries [6].

Secondary caries is a significant issue in dental practice, contributing to nearly 60% of restoration failures. Traditionally, such failures necessitate the replacement of restorations, a procedure that often results in the loss of additional healthy tooth structure and compromises the long-term integrity of the teeth. Furthermore, repeated restorative treatments impose a significant financial burden on healthcare systems [8]. Given that untreated or recurrent caries can ultimately lead to tooth loss, preventing such disease progression is critical for maintaining natural dentition and avoiding the need for complete edentulism management with subsequent implant-supported prostheses [9–11].

To address these challenges, the incorporation of antimicrobial agents into GIC has been explored as a strategy to enhance its therapeutic benefits. However, most modifications involving antibacterial agents, such as chlorhexidine (CHX), tend to compromise the physical and chemical properties of the cement [12–14]. Although CHX has been widely regarded as a gold-standard antimicrobial in dentistry, its use in GIC formulations raises concerns about adverse effects on bonding, as well as physical and mechanical performance [15]. Similarly, the use of antibiotics in GIC provides strong antibacterial effects but carries the risk of contributing to antibiotic resistance.

**Fig. 1:**
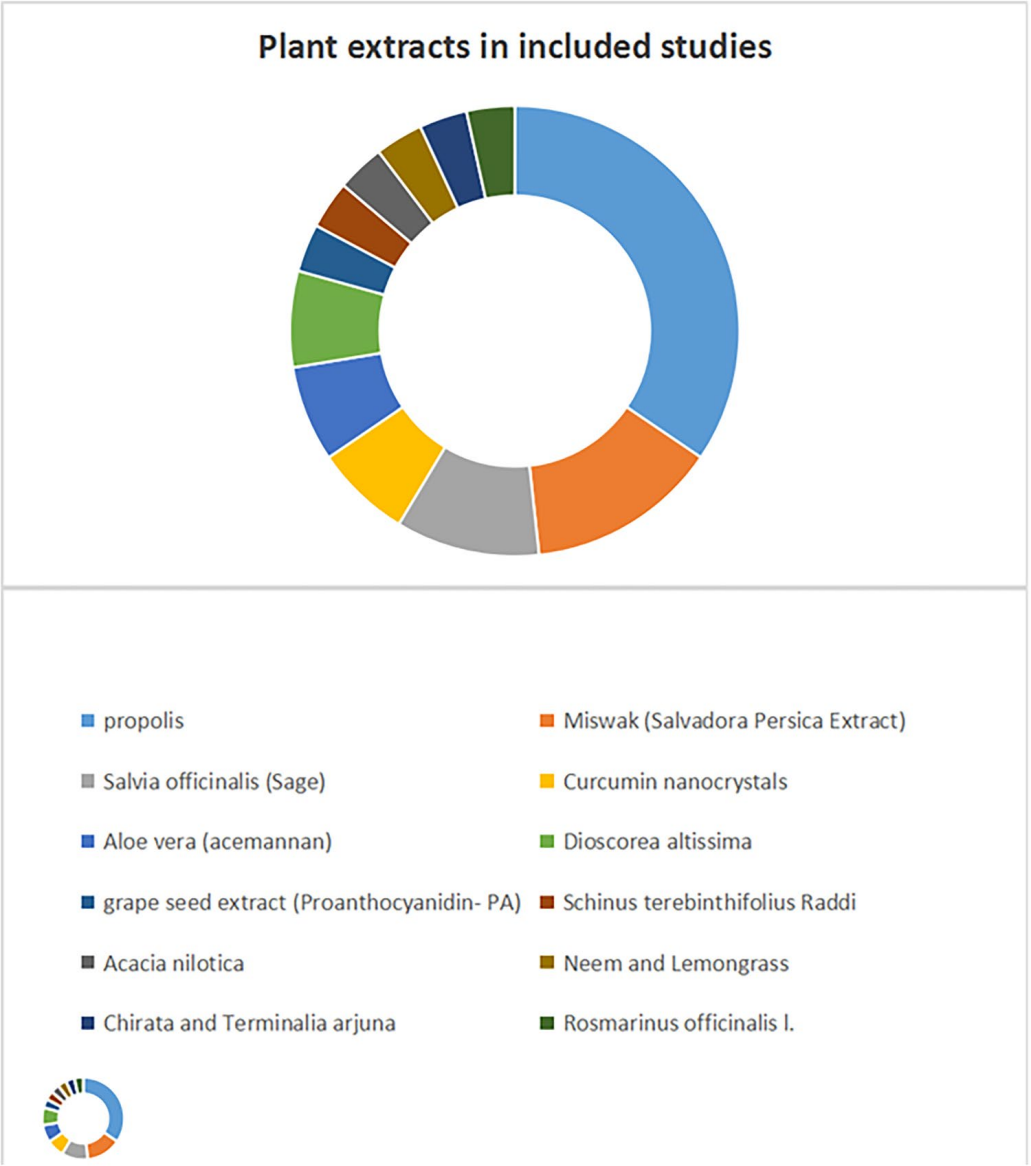
Pie chart of all plant extract incorporated in glass ionomer cement recently

In recent years, the exploration of natural and herbal antimicrobials has gained considerable attention. Plant-derived compounds, such as epigallocatechin-3-gallate (EGCG), have demonstrated promising antibacterial activity when incorporated into GIC [16]. Moreover, many plant extracts have been incorporated into GIC formulation such as Ethanolic Extract of Propolis (EEP)[17], *Miswak* or (*Salvadora Persica* Extract) [18], *Dioscorea altissima* [19], *Schinus terebinthifolius Raddi* [20], *Acacia nilotica* [21],*Salvia officinalis* [22], *Aloe vera* [23], *Neem and Lemongrass* [24] and *Rosmarinus officinalis* l. [25]. Among all studied plant extracts, Propolis extract was the most commonly used as shown in the pie chart **(**Fig. 1**).**

Propolis a natural resinous compound produced by honeybees* (Apis mellifera)*, commonly referred to as “bee glue”. It is collected from plant exudates such as resins, saps, and gums from buds, leaves, and bark of various plant species. Botanically, the composition of propolis varies depending on the local flora, climate, and geographic region. The bioactive compounds in propolis, such as flavonoids, phenolic acids, and aromatic esters, are largely influenced by the botanical origin. These compounds contribute to its well-documented antimicrobial, antioxidant, and anti-inflammatory properties, making propolis a valuable natural agent for various therapeutic and dental applications [26]. Its ability to inhibit cariogenic bacteria and other oral pathogens makes it an attractive candidate for dental applications [27, 28].

Furthermore, its antioxidant activity may contribute to improving the durability and longevity of restorative materials by mitigating oxidative stress and enhancing mechanical performance. Despite propolis’ complex bioactive composition, many of the included studies lacked detailed chemical characterization (e.g., FTIR, GC–MS, HPLC). This gap limits identification of the specific flavonoids or phenolic compounds responsible for the observed antimicrobial and mechanical enhancements. Incorporating propolis into GIC offers a promising approach to overcoming the dual challenges of bacterial resistance and suboptimal mechanical properties. This systematic review seeks to evaluate the effects of propolis incorporation into GIC on its antimicrobial, mechanical and other properties. By synthesizing current evidence, the review aims to identify the most effective formulations and concentrations of propolis, thus optimizing the clinical performance of GIC in contemporary dentistry.

## Methods

This systematic review was established following the guidelines provided by the Preferred Reporting Items for Systematic Reviews and Meta-Analyses (PRISMA). The research was pre-registered on the PROSPERO registration platform (ID: CRD42024627498).

### Eligibility criteria and search strategy

Studies were selected based on the following criteria as shown in Table 1:

**Table 1:** Inclusion and exclusion criteria

Inclusion criteria	Exclusion criteria
Literature in English language	Literature in a language other than English
Human clinical studies	Animal studies
In vitro studies	Letters to the editor, case reports, technical reports, cadaver studies, dissertations, incomplete trials, unpublished abstracts, reports, commentaries, and review papers
Studies comparing the physical or mechanical or surface mechanical or antibacterial or chemical properties of the *GIC* when modify it by any form of the propolis extractions in different concentrations	Studies discussing properties of only modified resin *GIC*
	Studies comparing previous properties for other plant extract

Using PICO format, the criteria included GIC as the population, Propolis as the intervention, conventional GIC as the comparator, and antimicrobial properties as the primary outcome. Mechanical properties, including compressive strength, shear bond strength, and microhardness as secondary outcomes. In vitro or in vivo studies were included from any publication year. Studies with missing full texts, irrelevant materials, focused on restorative materials other than GIC (e.g., composite, amalgam), alternative plant extracts, using plant extracts other than Miswak (e.g., Propolis, Neem), insufficient data, or poor quality were excluded.

Two separate authors systematically explored the indexed English literature by utilizing the following electronic databases: PubMed, Web of Science (core collection), Scopus, and the Cochrane library to gather articles published until the start of 2024. Additionally, the database search was supplemented with manual reference screening, ensuring that no key studies were overlooked. The PICO statement guided the search strategies employed. The articles were initially searched for in June 2024 and subsequently updated in October 2024. The search procedure employed combinations of Medical Subject Headings (MeSH) terms along with non-MeSH terms, combined with Boolean operators, to carry out the search successfully. The search was conducted using MeSH terms and Boolean operators. Detailed search strings are provided in Table 2**.**

**Table 2:** Details of search strings used for the systematic search

Database	Combination of Search Terms and Strategy	Article Numbers
MEDLINE-PubMed	((*GIC*s OR Glass-Ionomer Cement OR *GIC* OR GIC OR GICs) AND (propolis OR Bee Glue OR Glue, Bee OR Bee Bread OR Bread, Bee))	22
Scopus	((*GIC*s OR Glass-Ionomer Cement OR *GIC* OR GIC OR GICs) AND (propolis OR Bee Glue OR Glue, Bee OR Bee Bread OR Bread, Bee))	1
Web of Sciences (Core collection)	((*GIC*s OR Glass-Ionomer Cement OR *GIC* OR GIC OR GICs) AND (propolis OR Bee Glue OR Glue, Bee OR Bee Bread OR Bread, Bee))	31
Cochrane Library	((*GIC*s OR Glass-Ionomer Cement OR *GIC* OR GIC OR GICs) AND (propolis OR Bee Glue OR Glue, Bee OR Bee Bread OR Bread, Bee))	5

### Study selection and data extraction

All studies were uploaded into Rayan [29], where duplicates were manually screened and removed. The remaining articles were divided into two groups and assessed by four reviewers based on titles and abstracts, using the predetermined eligibility criteria. Each group was evaluated independently by two examiners. Discrepancies were resolved through group discussions to ensure agreement. Full-text screening was then conducted to finalize the included studies.

Duplicate articles were recognized and removed. Subsequently, the titles and abstracts of the other articles were thoroughly examined in line with the defined inclusion and exclusion criteria. Full-text evaluations were subsequently carried out on the narrowed-down articles, and the ultimate choice was cross-checked by another reviewer. Any conflicts concerning opposing articles were settled through conversations among the authors.

### Quality assessment method

The methodological quality and risk of bias of the included studies were independently assessed using the Modified CONSORT Scale [30, 31]. This tool comprises 14 items, each scored as “Yes” (criterion met) or “No” (criterion not met), with a maximum score of 14. Risk of bias was categorized as:

Low risk: 11–14 points.

Moderate risk: 7–10 points.

High risk: 0–6 points.

## Results

### Study selection and characteristics results

The systematic search across four databases yielded 59 articles. After removing duplicates (*n* = *31*) and screening titles and abstracts, 19 articles underwent full-text review. Of these, 18 studies met the inclusion criteria and were included in the qualitative and quantitative analysis. The studies primarily focused on the effects of incorporating ethanolic extract of propolis (EEP) into various commercial GIC brands, such as Fuji IX, Meron, and Ketac Cem, with concentrations ranging from 1 to 50%.

**Fig. 2:**
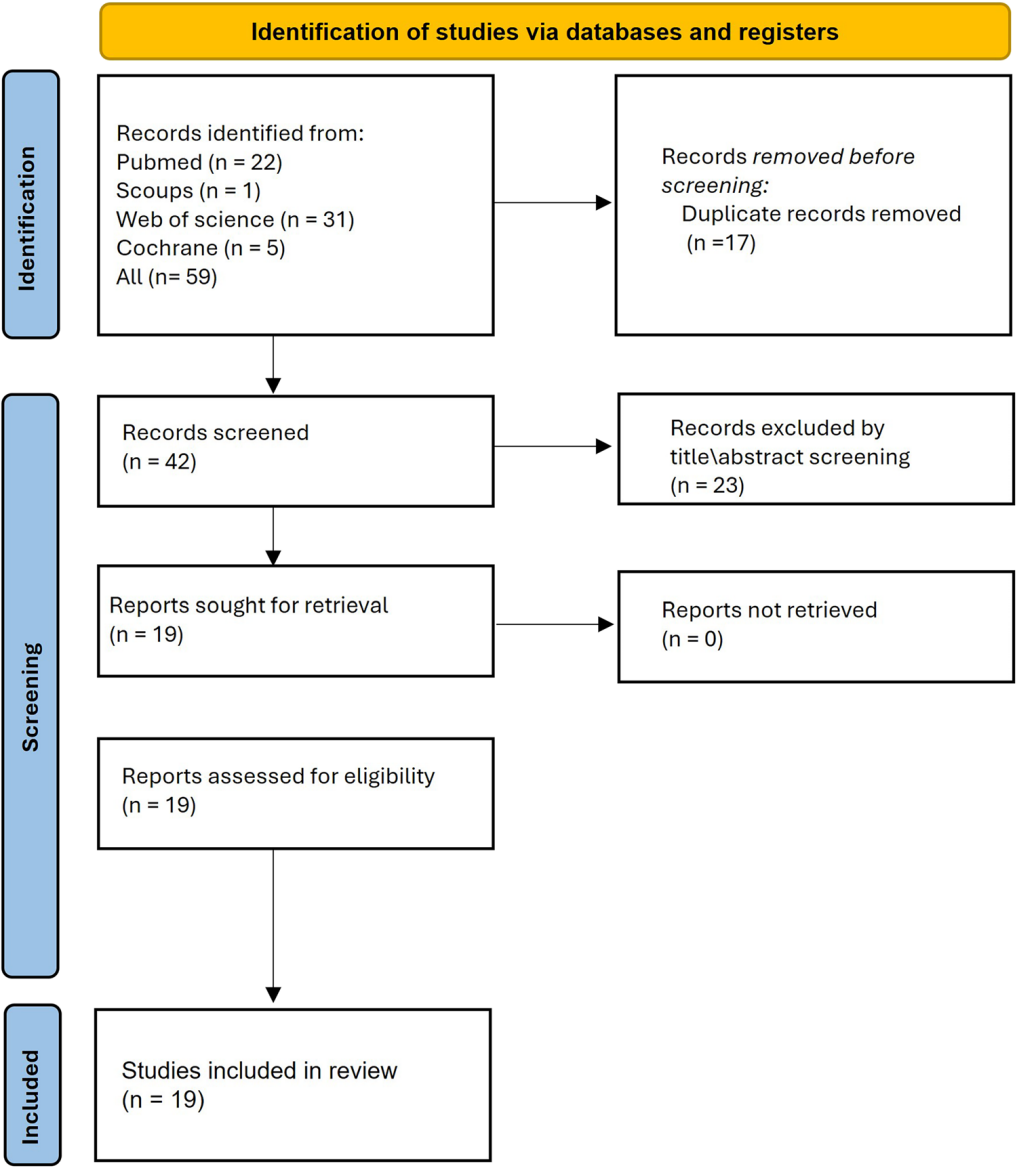
PRISMA Flow Diagram of Study Selection

A total of 18 studies were included in this systematic review after screening and applying the inclusion criteria. Pertinent information was arranged using specially crafted tables (Fig. 2).

These studies explored various aspects of propolis-modified GIC, including its antimicrobial efficacy, mechanical properties, and methods of preparation. A detailed summary of the study characteristics is presented in Table 3. This table outlines critical information about the included studies, such as the author, year, study design, aim of the study, sample details, GIC brand used, the source and extraction method of propolis, and how it was incorporated into the GIC. This comprehensive overview highlights the diversity in methodologies and experimental setups, which were considered during the synthesis of results (Table 3).

**Table 3:** Summary of study characteristics

Ref	Aim	Groups	GIC Brand	Propolis Source	Extraction Method	Propolis Form	Propolis Characterization	GIC Characterization	Addition to GIC	Key Findings
[32]	Assess antimicrobial, compressive strength, flexure strength, and bond strength	Unmodified PAC, 25MPAC, 50MPAC,	Fuji IX	Yucamiel (commercial)	Ethanolic extraction	Solution	___	FTIR, XRD	To mixed cement	Enhanced antimicrobial activity; reduced compressive and bending strength; unaffected shear bond strength
[33]	Evaluate microleakage and microhardness	Control, 10% EEP, 25% EEP, 50% EEP	Imicryl SC	Apis mellifera, Anatolia, Turkey	Ethanolic extraction	Thick paste	___	___	To GIC liquid	Increased antibacterial properties and microhardness; no adverse effect on microleakage
[34]	Compare physical–chemical, antibacterial, and compressive strength properties	GIC, PE (25 wt%), CHX	Maxxion C	Pharma Nectar, Brazil	___	___	flavonoid release	FTIR, SEM	To GIC powder	Improved compressive strength and antibacterial activity
[35]	Assess antimicrobial and physical properties	Fuji II, Fuji IX, 25% and 50% propolis	Fuji II, Fuji IX	Jahangir Pharmaceuticals, Iran	Aqueous extraction	Solution	___	___	To GIC liquid	No significant effects on antimicrobial properties or flexural strength
[36]	Evaluate antimicrobial activity, mechanical properties, and fluoride release	Control, RP10%, RP25%, RP50%	Meron, Riva	Paraíba, Brazil	Ethanolic extraction	Solution	___	___	To GIC liquid	Increased antimicrobial activity; no significant changes in mechanical properties or fluoride release
[37]	Investigate antibacterial efficacy and fluoride release	Various concentrations of propolis, CHX	Ionofil Plus	Egyptian supplier	Ethanolic extraction	Freeze-dried powder	___	___	To GIC powder	Synergistic effect on antimicrobial activity; enhanced fluoride release at higher propolis concentrations
[38]	Study antimicrobial activity of EEP-modified GIC	Control, 25% EEP, 50% EEP	Not reported	Imtenan, Egypt	Ethanolic extraction	Solution	Minimum inhibitory concentration (MIC)	___	To mixed GIC	25% EEP showed the best antibacterial action with the lowest concentration
[39]	Assess antibacterial and microshear bond strength	GIC, RMGI, with 10% and 25% EEP	Equia Forte	Imtenan, Egypt	Ethanolic extraction	Powder	___	___	To GIC liquid	Increased antibacterial effects; decreased microshear bond strength to dentin
[40]	Evaluate antibacterial and mechanical properties	Control, 10% EEP, 25% EEP, 50% EEP	___	Apis mellifera, Turkey	Ethanolic extraction	Solution	MIC	___	To GIC liquid	Increased antibacterial properties without negatively affecting mechanical properties
[41]	Evaluate mechanical properties, antibacterial effect, and in vivo biocompatibility	Meron, Ketac Cem, EEP-modified groups	Meron, Ketac Cem	Apis mellifera, Brazil	Ethanolic extraction	Solution	___	___	To mixed cement	Concentration-dependent antibacterial effects; no negative change in bond strength
[42]	Assess antimicrobial efficacy against S. mutans and L. acidophilus	GIC, GIC + Propolis, CHX, Chitosan	Fuji IX	HiTech Natural Products, India	Ethanolic extraction	Solution	___	___	To mixed cement	Propolis-modified GIC effective against S. mutans; CHX had higher antimicrobial activity overall
[43]	Assess the mechanical and antimicrobial properties of RMGIC	Control, 25% and 50% propolis	Fuji II LC	Not reported	Aqueous extraction	___	___	___	To mixed cement	No antibacterial activity; decreased flexural and bond strength
[44]	Assess antimicrobial activity of triphala and propolis-modified GIC	GIC, 50% EEP	Not reported	HiTech Natural Lab, Delhi	Ethanolic extraction	Solution	___	___	To mixed cement	Propolis-modified GIC provided significant antibacterial effects
[45]	Evaluate shear bond strength and fluoride release	Control, 1% EEP	Fuji IX	HiTech Natural Products, India	Ethanolic extraction	Sticky paste	___	___	To GIC liquid	Increased fluoride release; unaffected shear bond strength
[46]	Evaluate compressive strength	Control, 25%, 30%, 35% EEP	___	Trigona, Indonesia	Maceration in ethanol	Paste	Phytochemical screening (qualitative)	___	To mixed cement	Decreased compressive strength with EEP addition
[47]	Evaluate compressive strength and solubility	Control, 1% EEP	Fuji II	K-Link Health Products	Not reported	Solution	___	___	To mixed cement	Decreased compressive strength and increased solubility over 7 days
[48]	Evaluate antibacterial and antibiofilm properties	Control, 25% EEP, 50% EEP	Kavitan Pro	Turkey	Ethanolic extraction	Paste	___	___	To mixed cement	Significant antibacterial and antibiofilm efficacy against *S. mutans*
[49]	Investigate mechanical properties of GIC combined with propolis	Various formulations of GIC + propolis	KFL	Typified pure propolis	Lyophilization and ethanol	Solution, powder	___	___	To GIC liquid	Increased water sorption, solubility depended on formulation
[50]	Evaluate antibacterial effect of propolis-modified GIC	Control, 15% propolis, CHX	___	___	___	___	___	___	To GIC liquid	No significant antibacterial differences between propolis- and CHX-modified GIC.s

### Quality assessment results

Using the Modified CONSORT checklist for in vitro studies, the included studies generally demonstrated good methodological quality (Moderate risk) (Table 4).

**Table 4:** The quality analysis results

Item	[32]	[33]	[34]	[35]	[36]	[37]	[38]	[39]	[40]	[41]	[42]	[43]	[44]	[45]	[46]	[47]	[48]	[49]	[50]
1	1	1	1	1	1	1	1	1	1	1	1	1	1	1	1	1	1	1	1
2a	1	1	1	1	1	1	1	1	1	1	1	1	1	1	1	1	1	1	1
2b	1	1	1	1	1	1	1	1	1	1	1	1	1	1	1	1	1	1	1
3	1	1	1	1	1	1	1	1	1	1	1	1	1	1	1	1	1	1	1
4	1	1	1	1	1	1	1	1	1	1	1	1	1	1	1	1	1	1	1
5	0	0	0	0	0	0	0	0	0	0	0	0	1	0	1	0	0	1	0
6	0	0	0	0	0	0	0	0	0	0	0	0	0	0	0	0	0	0	0
7	0	0	0	0	0	0	0	0	0	0	0	0	0	0	0	0	0	0	0
8	0	0	0	0	0	0	0	0	0	0	0	0	0	0	0	0	0	0	0
9	0	0	0	0	0	0	0	0	0	0	0	0	0	0	0	0	0	0	0
10	1	1	1	1	1	1	0	1	1	1	1	1	1	1	1	1	1	1	1
11	0	0	0	0	0	0	1	0	0	1	1	1	1	1	1	1	1	1	0
12	1	0	0	1	0	0	0	1	0	1	1	1	1	1	1	1	1	1	1
13	1	0	0	1	0	1	1	1	0	0	1	0	0	0	0	0	0	1	0
14	0	0	0	0	0	0	0	0	0	0	0	0	0	0	0	0	0	0	0
Risk of bias score Score	8 Moderate risk	6 High risk	6 High risk	8 Moderate risk	6 High risk	7 Moderate risk	7 Moderate risk	8 Moderate risk	6 High risk	8 Moderate risk	9 Moderate risk	8 Moderate risk	9 Moderate risk	8 Moderate risk	9 Moderate risk	8 Moderate risk	8 Moderate risk	10 Moderate risk	7 Moderate risk

### Antibacterial effect results

#### *Streptococcus mutants *(*S. mutans*)

**Fig. 3:**
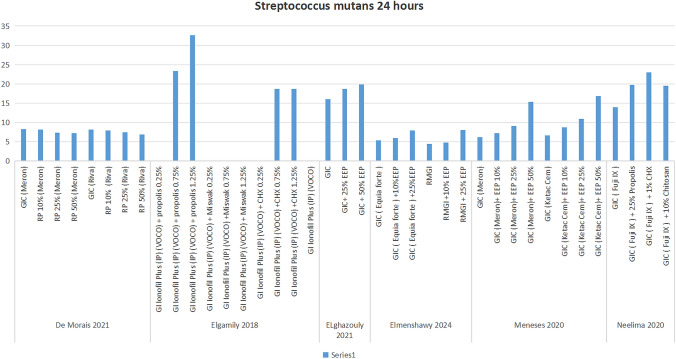
Antibacterial Effects on *S. Mutans* after 24h

Six studies [36–41, 46] investigated the antibacterial effect of propolis-modified GIC on *S. mutans* at the 24-h interval. Across all studies, propolis-modified GIC showed notably higher antibacterial activity than the control GIC, with effectiveness increasing alongside propolis concentration. *Elgamily *et al*.* [37] showed that, no difference was observed between unmodified GIC and GIC with 0.25% propolis, with both showing 0 mm in the disc diffusion test. Another study by *Meneses *et al*.* [41] compared two GIC brands (Ketac Cem and Meron), reported that both brands exhibited nearly three times the antibacterial activity when modified with 50% propolis. Specifically, Meron showed 6.2 mm in the agar diffusion test, increasing to 15.3 mm when modified, while Ketac Cem showed 6.6 mm, rising to 16.8 mm with 50% propolis [41]. In another study by *c*., although the concentration of propolis was unspecified, propolis-modified GIC again outperformed the control, with a GIC formulation modified with chlorhexidine (CHX) yielding even greater antibacterial activity [46] **(**Table 5**, **Fig. 3**)**.

**Table 5:** Summary of antimicrobial studies on *S. mutans*

Period	Code of the Bacteria	Name of the Test	Groups	Mean	SD	Refs
24 h	ATCC 25175	Colony-forming unit (CFU) assay	Control (Meron)	8.27	0.26	[36]
			RP10% (Meron)	8.17	0.23	
			RP25% (Meron)	7.38	0.13	
			RP50% (Meron)	7.23	0.14	
			Control (Riva)	8.19	0.25	
			RP10% (Riva)	7.96	0.22	
			RP25% (Riva)	7.42	0.08	
			RP50% (Riva)	6.89	0.17	
24 h	ATCC 25175	Agar diffusion assay	Glass ionomer + propolis 0.25%	23.30	4.43	[37]
			Glass ionomer + propolis 0.75%	32.64	2.26	
			Group 3: Glass inomer + propolis 1.25%	32.64	2.26	
			Group 4: Glass inomer + Miswak 0.25%	0.00	0.00	
			Group 5: Glass inomer + Miswak 0.75%	0.00	0.00	
			Group 6: Glass inomer + Miswak 1.25%	0.00	0.00	
			Group 7: Glass inomer + CHX 0.25%	0.00	0.00	
			Group 8: Glass inomer + CHX 0.75%	18.68	1.98	
			Group 9: Glass inomer + CHX 1.25%	18.68	1.56	
			Group 10: Glass inomer	0.00	0.00	
24 h	ATCC 25175	Agar diffusion assay broth microdilution test	Group 1: GIC	16	3.4	[38]
			Group 2: GIC + 25%EEP	18.7	3.7	
			Group 3: GIC + 50%EEP	19.9	5.4	
24 h			Group 1: GIC	5.38	0.96	[39]
			Group 2: GIC + 10%EEP	5.94	0.48	
			Group 3: GIC + 25%EEP	7.91	0.49	
			Group 4: RMGI	4.39	0.34	
			Group 5: RMGI + 10%EEP	4.8	0.22	
			Group 6: RMGI + 25%EEP	7.99	0.85	
24 h	ATCC-25175	Agar diffusion assay	Group 1: GIC (Meron)	6.2	0.5	[41]
			Group 2: Meron + EEP10%	7.2	1.0	
			Group 3: Meron + EEP25%	9.1	0.6	
			Group 4: Meron + EEP50%	15.3	1.0	
			Group 5: GIC (Ketac Cem)	6.6	0.4	
			Group 6: Ketac Cem + EEP10%	8.7	0.6	
			Group 7: Ketac Cem + EEP25%	10.9	0.8	
			Group 8: Ketac Cem + EEP50%	16.8	0.7	
24 h	MTCC 497	Agar diffusion assay	GIC	13.90	2.38	[42]
			GIC with Propolis	19.70	2.67	
			GIC with CHX	23.00	3.43	
			GIC with Chitosan	19.50	2.68	
48 h	ATCC 25175	Agar diffusion assay	GIC	17.5	0.33	[34]
			PE (25 wt%)	25.0	0.35	
			20 μl of PE	25.0	0	
			20 μl of (CHX)	17.3	0.33	
48 h	ATCC 25175	Agar diffusion assay	GIC with aqueous extract of triphala	11.60	0.41	[44]
			GIC with 50%EEP	11.80	1.15	
			GIC	5.50	0.50	
42 h	ATCC 25175	Agar diffusion assay MIC (broth microdilution test) Biofilm Assay	GIC	0.3	0.1	[48]
			25% EEP group	0.7	0.1	
			50% EEP group	0.8	0.1	
7 days	MTCC 497	Agar diffusion assay	GIC	12.60	2.12	[42]
			GIC with Propolis	18.50	2.51	
			GIC with CHX	17.60	2.76	
14 days	MTCC 497	Agar diffusion assay	GIC	11.70	1.45	[42]
			GIC with Propolis	16.50	2.22	
			GIC with CHX	19.30	2.87	
30 days	ATCC-25175	Agar diffusion assay	GIC (Meron)	5.3	0.2	[41]
			Meron + EEP 10%	5.7	0.6	
			Meron + EEP 25%	11.9	1.0	
			GIC (Ketac Cem)	5.8	0.9	
90 days	ATCC-25175	Agar diffusion assay	GIC (Meron)	6.8	0.7	[41]
			Meron + EEP 10%	7.1	0.6	
			Meron + EEP 25%	13.0	0.8	
			GIC (Ketac Cem)	6.9	1.1

At the 48-h interval, two studies confirmed that propolis-modified GIC remained more effective than the unmodified GIC [34, 43] (Table 5). One of the two studies tested at 42 h, reported consistent findings [48]. Another study extended the assessment to 7 and 14 days, again showing that propolis-modified GIC sustained its superior antibacterial effect. A Study also examined long-term effects at 30 and 90 days, confirming the continued efficacy of propolis-modified GIC. Here, Ketac Cem modified with propolis showed notably better results compared to Meron modified with propolis [41] (Table 5).Propolis-modified GIC consistently outperformed the control GIC in inhibiting *S. mutans*, with higher concentrations of propolis producing stronger antibacterial effects. The 50% propolis-modified GIC demonstrated the highest efficacy, followed by 25% and 10% concentrations.

#### Lactobacillus

Two studies [43, 46] assessed the antibacterial effect of propolis-modified GIC on *Lactobacillus*. *Azalia *et al*.* [46] examined it at 24 h, 7 days, and 14 days, consistently showing that propolis-modified GIC performed slightly better than the control GIC at each interval. A study by *Panahandeh *et al. [43] evaluated the effect after 48 h using the agar disk diffusion test, where GIC with 50% ethanol-extracted propolis EEP produced an inhibition zone nearly double that of the control group, with a mean of 11.9 mm for the modified group compared to 5.1 mm for the control (Table 6).

**Table 6:** Summary of antimicrobial studies on *Lactobacillus*

Period	Code of the Bacteria	Name of the Test	Sample Size	Groups	Refs	Mean	SD
24 h	MTCC 10307	Agar diffusion test	10	GIC	[42]	12.40	± 0.97
				GIC with Propolis		13.70	± 0.68
				GIC with CHX		20.30	± 2.00
				GIC with Chitosan		16.50	± 0.98
48 h	ATCC 4356	Agar diffusion test	5	GIC with aqueous extract of triphala	[44]	11.70	0.83
				GIC with 50%EEP		11.90	0.65
				GIC		5.10	0.74
7 days	MTCC 10307	Agar diffusion test	10	GIC	[42]	10.30	± 0.94
				GIC with Propolis		11.70	± 0.68
				GIC with CHX		16.20	± 2.04
				GIC with Chitosan		12.50	± 0.98
14 days	MTCC 10307	Agar diffusion test	10	GIC	[42]	8.40	± 0.97
				GIC with Propolis		9.70	± 0.68
				GIC with CHX		18.30	± 2.00
				GIC with Chitosan		14.50	± 0.98

#### Candida albicans

*Andrade *et al*.* [34] assessed the antibacterial effect of propolis-modified GIC on Candida albicans using the Colony-Forming Unit (CFU) assay at 24 h, found that 25% propolis-modified GIC with Meron yielded 5.38 CFU compared to 7.32 for the control, while 50% propolis-modified GIC with Riva scored 6.29 CFU versus 7.08 for the control. At 48 h, the agar diffusion method, where 20 μl of propolis extract produced the largest inhibition zone, followed by 25% propolis-modified GIC at 21 mm, outperforming the control at 15 mm (Table 7**)**.

**Table 7:** Summary of antimicrobial studies on *Candida albicans* at 24 and 48 Hours

Period	Code of the Bacteria	Name of the Test	Sample Size	Groups	Refs	Mean	SD
24 h	ATCC 90028	Colony-forming unit (CFU) assay	3	Control (Meron)	[36]	7.32	0.42
				RP10% (Meron)		6.90	0.42
				RP25% (Meron)		5.38	0.55
				RP50% (Meron)		5.47	1.09
				Control (Riva)		7.08	0.60
				RP10% (Riva)		6.52	0.06
				RP25% (Riva)		6.32	0.17
				RP50% (Riva)		6.29	0.45
48 h	ATCC 18804	Agar diffusion method	3	GIC	[34]	15.00	0.50
				PE (25 wt%)		21.00	0.55
				20 μl of PE		30.00	0.00
				20 μl of (CHX)		12.00	0.00

#### Streptococcus salivarius

**Fig. 4:**
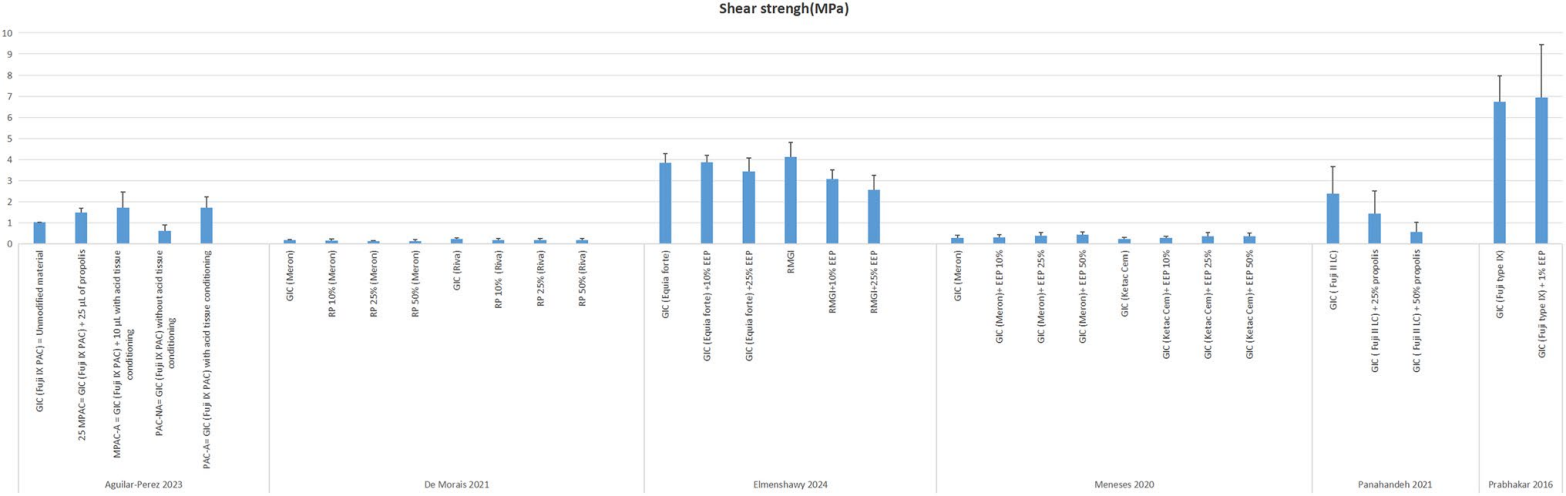
The results of shear bond strength

Only one study [34] examined the antibacterial effect of propolis-modified GIC on *Streptococcus salivarius*. At 48 h, both the control group and 25% propolis-modified GIC produced identical inhibition zones of 20 mm in the agar diffusion test. Propolis-modified GIC demonstrated enhanced antibacterial effects across multiple bacterial strains, particularly at higher concentrations, with 50% propolis showing the most substantial impact (Fig. 4).

### Mechanical properties results

The mechanical properties of propolis-modified GIC varied significantly based on the concentration of propolis and the type of GIC used:

#### Bond strength

Six studies [32, 36, 39, 41, 43, 45] assessed shear bond strength with mixed outcomes. Four studies [32, 39, 41, 45] reported that adding propolis increased shear bond strength compared to GIC alone, with optimal concentrations ranging from 1 to 50%. Notable improvements were reported at concentrations of 10% [39], 25% [32], and 50% [33], with the highest increase attributed to the 50% formulation. Slight improvements were also noted with 1% ethanolic extract [45].

In contrast, other findings indicated that adding propolis could reduce bond strength [36, 43]. Unmodified GIC exhibited the best performance in one analysis [36], followed by formulations with 10% and 50% propolis. Another study reported a significant reduction in bond strength at a 50% concentration, which decreased the value to approximately one-fourth of the control group’s strength [43]. These variations highlight the concentration-dependent effects of propolis on the bonding performance of GIC.

##### Compressive strength

The effect of propolis-modified GIC on compressive strength was evaluated in four studies [32, 36, 46, 47], with mixed results. One investigation found that incorporating 25% propolis significantly enhanced compressive strength compared to unmodified GIC, while a 50% concentration led to a marked reduction [36]. The same study tested a different GIC brand, where 25% again demonstrated the best results, but the difference between GIC and 50% was minimal [36].

**Fig. 5:**
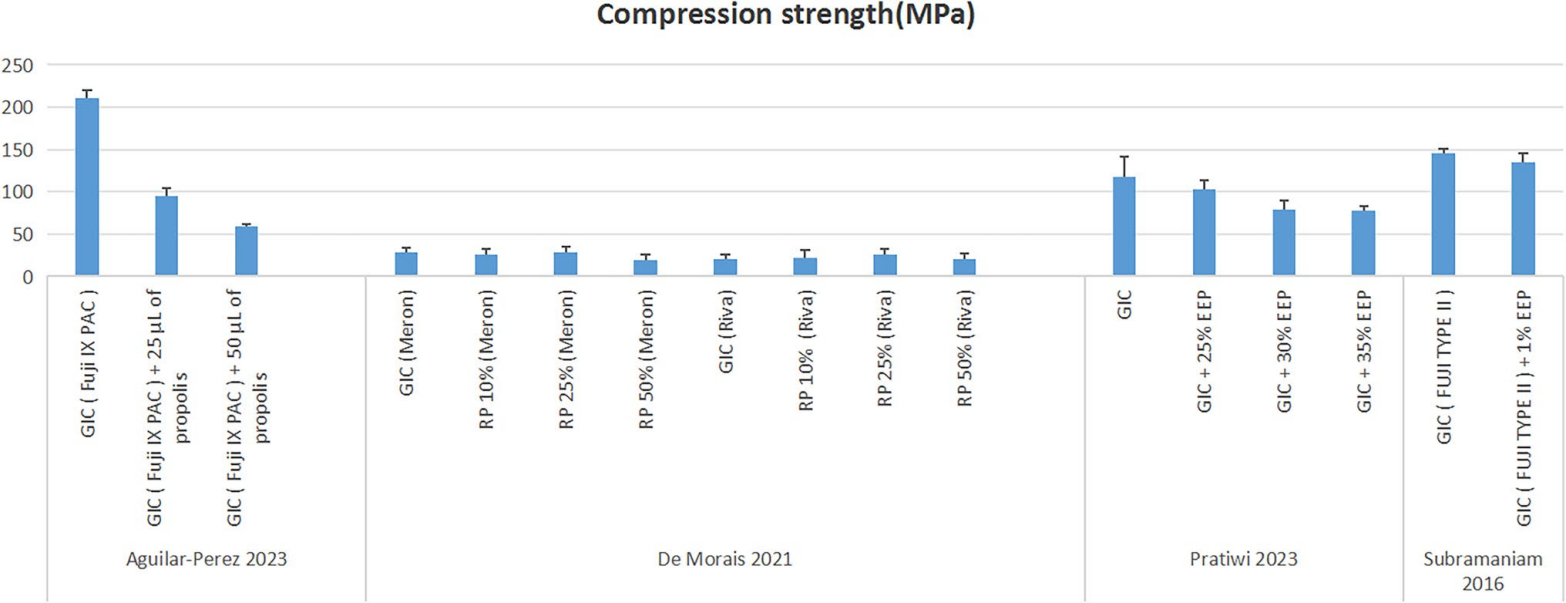
Compressive Strength Results

In contrast, other analyses indicated a reduction in compressive strength with propolis addition [32, 46, 47]. One study showed that unmodified GIC exhibited the highest strength, with 50% propolis reducing it to about one-third of the control value [32]. Another found unmodified GIC to outperform all tested concentrations, including 25%, 30%, and 35%, although the difference between unmodified GIC and 25% propolis was minimal [46]. A further evaluation revealed only a slight advantage of unmodified GIC over 1% propolis. These findings highlight the varying effects of propolis on compressive strength, depending on concentration and formulation [47] (Fig. 5**).**

##### Flexural strength

**Fig. 6:**
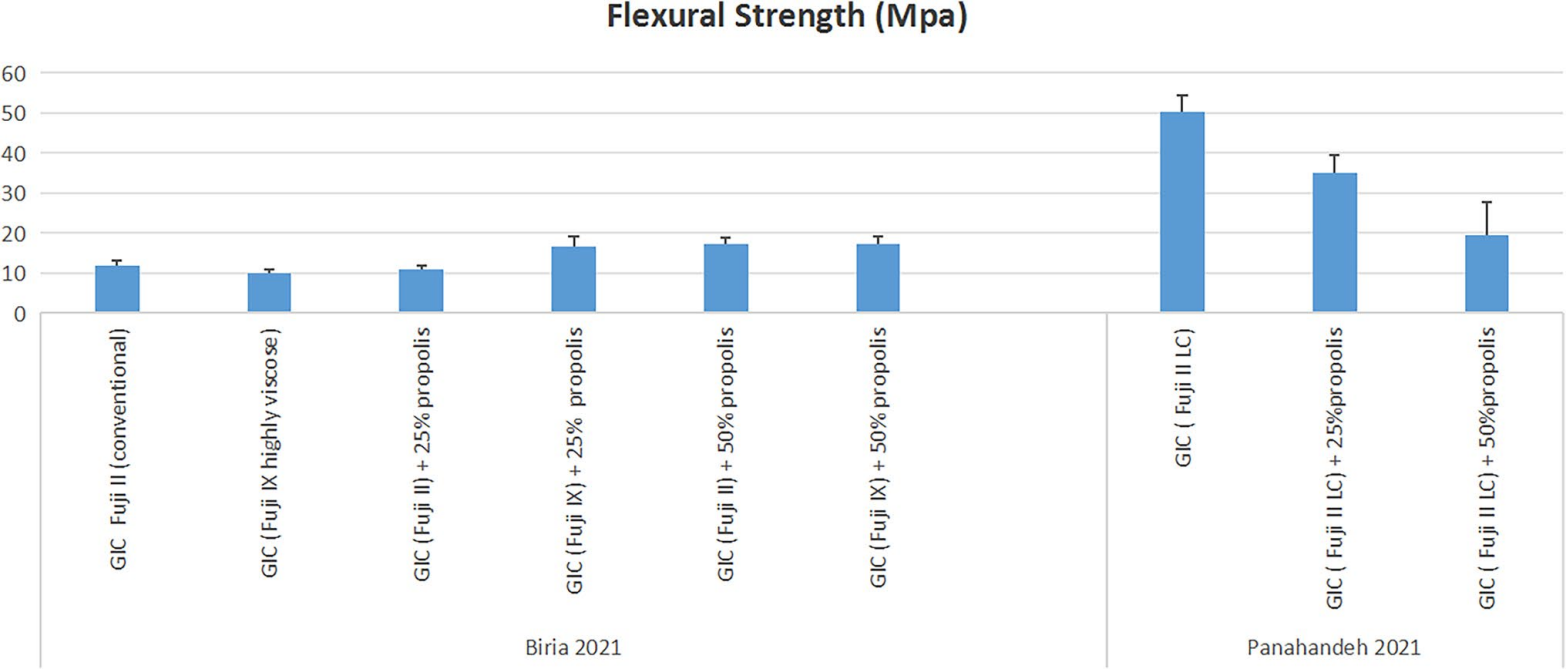
Flexural Strength Results

Two studies evaluated flexural strength [35, 43], a study reported that high-viscosity GIC (e.g., Fuji IX) better tolerating propolis incorporation than low-viscosity GIC (e.g., Fuji II) [43]. in low-viscosity GIC, the control group exhibited the highest strength, followed by 25% and 50% propolis-modified GIC, with slight differences. In high-viscosity GIC, the 25% propolis group showed the best strength [43]. In contrast, the other study reported that the GIC control group had significantly higher strength than both the 25% and 50% propolis-modified formulations, with the control group being approximately 1.5 times stronger than the 50% propolis group [43] (Fig. 6**)**.

##### Microhardness

**Fig. 7:**
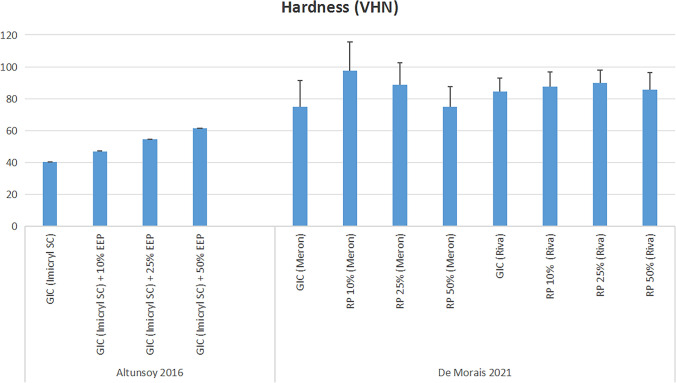
Microhardness Results

Two studies [33, 36] reported a concentration-dependent improvement in microhardness. One study reported that 50% propolis exhibited the highest hardness, followed by 25%, 10%, and GIC, with the 50% formulation being nearly three times harder than the GIC control group [33]. The other study found that, with the Meron GIC brand, 10% propolis had the best hardness, followed by 25%, GIC, and 50%, with minimal differences. When using the Riva GIC brand, 25% propolis demonstrated the highest hardness, followed by 10%, 50%, and the GIC control group [36] (Fig. 7**)**.

##### Diametral tensile strength

**Fig. 8:**
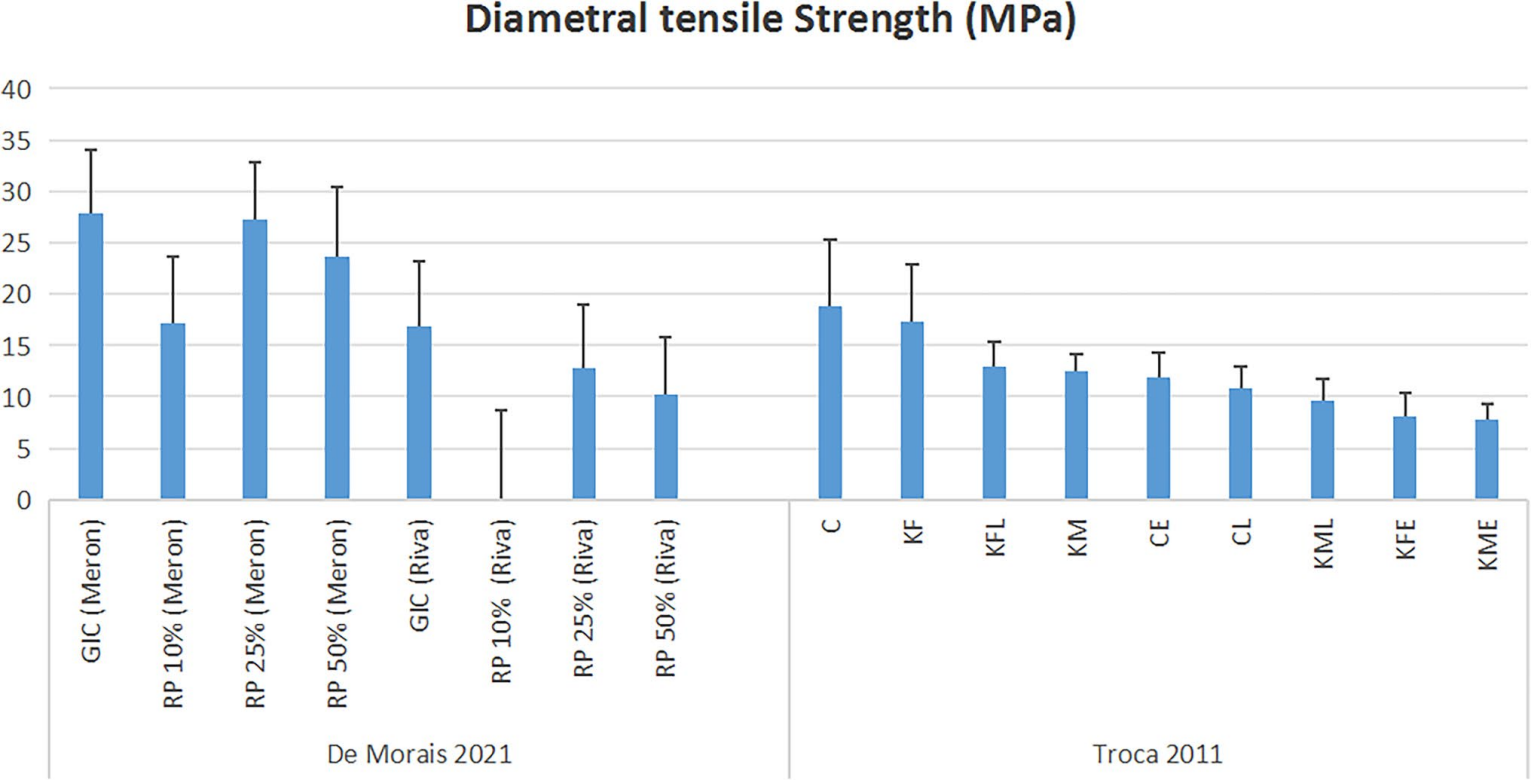
Diametral Tensile Strength results

Two studies evaluated diametral tensile strength [36, 49]. A study found that, with the Meron GIC brand, GIC alone exhibited the highest strength, followed by 25%, 50%, and 10% propolis. When using the Riva GIC brand, GIC again demonstrated the best strength, followed by 10%, 25%, and 50% propolis [36]. The other study tested different brands, reporting that Brand ChemFlex had the highest tensile strength, followed by Brands Ketac Fil Plus and lyophilized Ketac Fil Plus [49] (Fig. 8**).**

### Others

#### Fluoride release

Three studies the fluoride release [36, 37, 45] *Prabhakar *et al. [45] measured the initial fluoride release where propolis modified GIC exhibited a significantly higher mean fluoride release of 10.96 ± 2.01 ppm after 24 h compared to conventional GIC of 5.42 ± 0.46 ppm on the first day. This difference was statistically significant (*p* < *0.001*). Fluoride release overtime was also measured where conventional GIC showed a decline in fluoride release from 5.42 ± 0.46 ppm on the first day to 0.55 ± 0.04 ppm by the seventh day.

The propolis modified GIC also demonstrated a decrease in fluoride release, dropping from 10.96 ± 2.01 ppm after 24 h to 5.24 ± 1.03 ppm by the seventh day. Also, *Elgamily *et al*.* [37] reported that the incorporation of 0.25%, 0.75% and 1.25% into GIC elevated the release of fluoride at 6 h measuring time. On the other hand*, De Morais *et al*.* [36] recorded no statistically significant differences between different concentrations of propolis modified GIC and the conventional (*P* > *0.05*).

#### Microleakage

Microleakage surrounding dental restoration materials is a significant issue in clinical dentistry. It can be described as the clinically imperceptible movement of bacteria, fluids, molecules, or ions between a cavity wall and the restorative materials used on it [51]. *Elgamily *et al*.* [37] evaluated the effect of EEP added to GIC on microleakage, using a dye penetration method to assess the degree of leakage in standard Class II cavities filled with GIC containing varying concentrations of EEP. The results indicated no statistically significant differences in microleakage among the different groups tested, suggesting that EEP did not adversely affect the sealing ability of GIC.

#### Aesthetics

Propolis-modified GIC displayed a yellowish discoloration proportional to the concentration of propolis, limiting its use in aesthetic restorations. The yellow color alteration of GIC linked to EEP might not be an issue when used as a base or liner, but in the anterior area, it could have a detrimental impact on aesthetics [33]. The plant extract level of purity plays a crucial role in determining its effects on GIC. Crude extracts may contain impurities that could interfere with the setting reaction or compromise the mechanical properties and even aesthetics. A study confirmed that purification of *Salvadora Persica Extract* (SPE) is a factor that requires additional examination, since the brownish oil utilized in this research was not a pure material [52].

#### Handling properties

The handling characteristics, such as setting time and viscosity, were minimally affected by low-to-moderate concentrations (10%–25%) but required adjustment at higher concentrations (50%) [46].

## Discussion

This systematic review evaluated the effects of incorporating propolis into GIC on its antimicrobial and physico-mechanical properties. Insights from the included studies revealed significant variations depending on the type of GIC, concentration of propolis, and extraction methods.

### Narrative heterogeneity analysis

The included studies exhibit significant heterogeneity in several methodological domains, including:

#### Variability in GIC Brands

Differences in performance metrics were observed across brands (e.g., Fuji IX, Meron, Ketac Cem), emphasizing the need for brand-specific evaluations. *De Morais et. al* [36]*.* used two different orthodontic GIC, Meron (VOCO, Cuxhaven, Cuxhaven, Germany) and Riva (SDI, Bayswater,Victoria, Australia). *Meneses *et al*.* [41] used Meron (Voco, Cuxhaven, Germany) and KetacCem (3M/ESPE, Seefeld, Germany). *Troca *et al*.* [49] used Ketac Molar Easymix (3M ESPE, Dental Products, St. Paul, MN, USA), ChemFlex© (C) (DENTSPLY, Rio de Janeiro, RJ, Brazil) and Ketac Fil Plus (3M ESPE, Dental Products, St. Paul, MN, USA).

#### Propolis source

It is important to note that the source and preparation of the propolis extract varied across studies; some authors utilized commercially available propolis extracts [32, 34, 42, 45, 47], while others prepared the extracts themselves [33, 35–41, 43, 44, 46, 48, 49]. Commercial plant extracts are standardized for concentration and composition, making them readily accessible for research and clinical use. However, they often contain additives or preservatives that can interfere with antimicrobial properties [53]. Also, suppliers may not fully disclose extraction methods, leading to uncertainties about the purity and bioactive potential of the extracts [2, 53]. In contrast, researchers often choose to prepare their own plant extracts to maximize the desired phytochemicals, which may improve their antimicrobial and therapeutic effects against oral pathogens [2, 53].

#### Extraction methods

The successful incorporation of plant extracts into GIC depends heavily on the extraction method employed, as it influences the concentration and purity of the bioactive compounds. These methods are selected based on their efficiency in isolating the desired phytochemicals while maintaining their stability and bioactivity. Most studies employed ethanolic extraction [32, 36–42, 44–46, 48, 49] with variations in temperature and time. Others used aqueous extracts [33, 35, 43]. In all mentioned studies solvent extraction (ethanolic or aqueous) was utilized that involves soaking plant material in a solvent such as ethanol, methanol, or acetone, chosen based on the solubility of the target compounds. The process is versatile, allowing for the extraction of a wide range of bioactive compounds. After extraction, the mixture is filtered to separate the liquid extract, which is rich in bioactive components like flavonoids and phenols. This method is widely used due to its efficiency in isolating active ingredients for enhancing the antimicrobial and mechanical properties of GIC [54]. Other extraction methods could be further utilized such maceration [55–57], soxhlet extraction [58, 59], hydrothermal Extraction [60, 61], and decoction [62]. Also, several advanced extraction techniques are not yet widely applied like Ultrasonic-Assisted Extraction (UAE) [63], Supercritical Fluid Extraction (SFE) [64], Microwave-Assisted Extraction (MAE) [65], and Hydrodistillation [66].

#### The form of propolis after extraction

Plant extracts can be utilized into several forms, including solutions, powders, and lyophilized extracts. Some studies utilized the extract as solutions [32, 35, 36, 38, 40, 41, 47, 49] while others added it as powders [37, 39], and others added it as a paste [33, 45, 46, 48]. Solutions are typically alcohol or water-based, allowing for easy mixing with GIC components and ensuring uniform distribution within the cement matrix. However, the choice of solvent can affect the solubility and stability of bioactive compounds. Powders, obtained by drying and grinding plant materials or through freeze-drying, offer flexibility in dosage and ease of mixing. They are generally more stable during storage, but the particle size and consistency can influence the final properties of the modified GIC, such as setting time and mechanical strength [39, 67]. A study utilized propolis extracts in both liquid and powder forms. The ethanolic extract of propolis (EEP) was incorporated as a liquid by adding it to the GIC liquid component using a pipette. In contrast, the lyophilized form of propolis was used as a powder, which was mixed with the GIC powder component. Eventually, the GIC modified with the lyophilized form of propolis extract allowed for greater water sorption when compared to those modified by EEP. This allowed the researchers to investigate the effects of propolis on GIC in different physical forms [68].

#### Concentration

The concentration of propolis incorporated into GIC plays a critical role in determining its mechanical strength, antimicrobial activity, and biocompatibility. Various studies have explored different concentrations to optimize these properties. For instance, *Altunsoy *et al*.* [33] investigated the effects of EEP at concentrations of 10%, 25%, and 50% in GIC. The study found that there were no statistically significant differences among the groups in terms of microleakag. However, higher concentrations (25% and 50%) led to an increase in the VHN values, with 50% recording the highest microhardness. This suggests sometimes that higher concentrations may be more suitable for maintaining the structural integrity of GIC while still harnessing the beneficial properties of the plant extract [52]. Similarly, *De Morais *et al. [36] explored the use of EERP at varying concentrations in GIC. The study concluded that higher extract concentrations enhanced the antimicrobial activity against *S.mutans* but could compromise the compressive strength of the material. This highlights the delicate balance required in determining the optimal concentration, as higher amounts may improve antimicrobial efficacy at the cost of mechanical strength [2]. These findings underscore the importance of carefully selecting and optimizing the concentration of plant extracts in GIC. The goal is to achieve a formulation that maximizes the antimicrobial and bioactive benefits of the plant extracts while maintaining or enhancing the material’s mechanical properties. Table 3 provides a detailed overview of the concentrations used in various studies, highlighting the outcomes and implications for GIC performance.

#### Methods of propolis addition to GIC

Direct addition**,** the most straightforward method involves the direct addition of plant extracts into GIC. Some studies added Propolis extract to the powder of GIC [34, 37], For instance, in a study by *Altunsoy *et al., ethanolic extracts of propolis were incorporated into the GIC powder. This method preserved the handling characteristics of the liquid while enhancing the material’s microhardness without significantly altering microleakage [33]. Similarly, *El-Tatari *et al*.* found that incorporating *Salvadora persica* extract into the powder component helped maintain the desired physical properties at lower concentrations [52]. On the other hand other studies added Propolis to the liquid [33, 35, 36, 40], *Troca *et al*.* added EEP to the GIC liquid, which led to increased water sorption and decreased diametral tensile strength suggesting that while the addition to the liquid component can enhance certain bioactive properties, it may also introduce challenges related to the material’s stability and mechanical integrity [68]. *Panahandeh *et al*.* [43] explored the effect of adding propolis to the resin-modified GIC liquid, finding that it did not significantly improve the antibacterial properties but did negatively impact the flexural and shear bond strengths. This highlights the potential drawbacks of modifying the liquid component, especially in formulations where the liquid plays a critical role in the material’s overall performance. A few studies introduced plant extracts into the already mixed cement [15, 42, 43, 47]**.** Although this method requires careful consideration to avoid adverse effects on the setting process [18, 23, 47].Ultimately, the choice of whether to add the plant extract to the powder or liquid component of GIC depends on the desired balance between mechanical properties and bioactivity. Table 3 summarizes the various methods of incorporating plant extracts into GIC, detailing whether they were added to the powder or liquid components and the resulting impacts on the material’s performance.

Rather than direct addition, there are many methods of incorporation that should be considered in modifying GIC. Several studies have explored the impact of surface coatings on the performance of GIC [69, 70]. Further research evaluated the mechanical properties of GIC coated with G-Coat Plus, finding that the coated samples exhibited enhanced compressive and flexural strength compared to uncoated samples [71]. Also, Encapsulation offers a more sophisticated approach where plant extracts are encapsulated within nanoparticles or microspheres [72, 73]. This technique not only allows for a controlled release of the bioactive compounds but also minimizes any negative impact on the setting reaction and mechanical properties of the cement [73]. Using plant extracts in the green synthesis of nanoparticles is an exciting development within the encapsulation method. The bioactive compounds from plant extracts are utilized to create nanoparticles that serve as protective carriers. These nanoparticles can then be incorporated into GIC, facilitating a controlled release of the plant-derived agents. This approach not only enhances the antimicrobial properties of GIC but also aligns with eco-friendly practices in material science. This holds a promising approach that combines sustainability with advanced material science. Further studies are needed to optimize these synthesis processes and explore the full range of applications for propolis-derived nanoparticles. Several studies have utilized propolis in the green synthesis of nanoparticles such as silver [74],Gold [75], ZnO [76], Copper [77] and Titanium Dioxide Nanoparticles [78], highlighting significant antimicrobial activities.

Also, combining propolis with other antimicrobials like Chitosan-propolis combinations demonstrate significant potential in dental applications, particularly for preventing cariogenic bacteria [79, 80]. Additionally, chitosan-based propolis varnishes (5%, 10%, and 15%) exhibit strong antimicrobial activity, comparable to chlorhexidine varnishes, with rapid film formation, excellent tooth surface adherence, and sustained release properties, making them suitable for clinical use in caries prevention. These formulations leverage the natural antimicrobial properties of chitosan and propolis, offering an effective, biocompatible alternative to traditional dental treatments [81], as well as substitutes for industrial antibiotics [82]. Also, EEP, rich in polyphenols and flavonoids, exhibit broad-spectrum bioactivities and synergistically enhance the efficacy of antibiotics, particularly those targeting cell wall synthesis, such as vancomycin and oxacillin [83]. The combination of propolis extract with calcium hydroxide demonstrated enhanced biological effects compared to calcium hydroxide alone as a pulp capping bioactive material [84].

While studies have demonstrated the potential benefits of incorporating Propolis into GIC formulations, the specific application of these extracts such as surface coatings, encapsulation in green synthesis of nanoparticles and in combination with other bioactive agents have not been documented in the literature to be applied in GIC formulations. This presents an opportunity for future research to explore the feasibility and efficacy of using plant extracts in this innovative way, potentially enhancing the performance of GIC in clinical applications.

#### Variation in methodological assessment

For antimicrobial testing many methods were reported.

Agar Diffusion Method

The agar diffusion method is widely used for evaluating antimicrobial activity; however, it has limitations such as variability in diffusion rates and dependence on agar composition, which can lead to inconsistent results across studies. This variability is noted in studies that emphasize the need for standardized protocols to ensure reproducibility in antimicrobial testing [85].Research has shown that the size of the inhibition zone produced by this method may not accurately reflect the true antimicrobial potency, as it only provides qualitative data rather than quantitative measurements of minimum inhibitory concentration (MIC) [86].

Microdilution Method

The microdilution method is considered more precise for determining MIC values, but it is sensitive to environmental factors such as pH and nutrient availability, which can influence bacterial growth and skew results [86, 87]. Studies indicate that this method requires careful standardization of inoculum size and incubation conditions to avoid variability, as discrepancies in these parameters can lead to misleading conclusions about the efficacy of antimicrobial agents [87].

Comparative analyses have demonstrated that different methodologies can yield divergent results regarding the effectiveness of antimicrobial agents. For instance, a study comparing agar diffusion and microdilution methods found significant differences in the reported MIC values for certain plant extracts, highlighting the importance of methodological choice in interpreting antimicrobial efficacy [88]. Additionally, research has pointed out that natural compounds like propolis may exhibit complex interactions within different testing environments, which may not be fully captured by either method alone [89].

The choice of methodology should align with the specific research question and desired outcomes. While agar diffusion provides a quick assessment of antimicrobial potential, microdilution offers a more detailed understanding of how concentrations affect bacterial growth inhibition. It is crucial for researchers to report their methodologies transparently and consider potential biases introduced by their chosen techniques when interpreting results related to antimicrobial efficacy.

These methodological differences, combined with the lack of standardized propolis characterization (e.g., FTIR, GC–MS, HPLC analyses), limit direct comparability and preclude meta-analytic synthesis. Future studies should adopt unified protocols for both material preparation and bioactive component identification to enable meaningful comparisons.

### Methodological quality and bias considerations

Although most included studies demonstrated good reporting on intervention details, many failed to meet key methodological criteria such as randomization, blinding of operators and assessors, and sample size calculation. The absence of these measures introduces risk of selection and performance bias, reduces reproducibility, and undermines the external validity of findings. Without these safeguards, the observed effects of propolis modification may be overestimated or under-representative of clinical outcomes.

### Chemical characterization gap

A notable limitation across the reviewed literature is the lack of detailed chemical characterization of propolis extracts. Techniques such as FTIR, GC–MS, and HPLC can identify and quantify key flavonoids, phenolic acids, and esters responsible for antimicrobial and mechanical effects. Without such analyses, it remains unclear which specific bioactive compounds drive the observed benefits, and batch-to-batch variability in propolis composition may significantly influence results.

#### Propolis extract characterization

The characterization of propolis extract is crucial to understanding its bioactive components and their potential effects when incorporated into GIC. Various methods have been employed to analyze the extract. As highlighted in Table 3 studies overlooked characterization of the propolis while there are many methods of characterization such as traditional phytochemical screening that is used to identify and characterize the bioactive compounds present in plant extracts, such as alkaloids, flavonoids, saponins, phenolic compounds and terpenoids supporting its incorporation into GIC to enhance antimicrobial efficacy and overall performance [46]. Other characterization methods used for chemical characterization of plant extracts like High-Performance Liquid Chromatography (HPLC) which is used to identify and quantify flavonoids, phenolic acids, and other bioactive compounds in propolis extracts [20, 68]. Also, Gas Chromatography-Mass Spectrometry (GC–MS) was employed for volatile compound analysis, providing insights into the chemical composition of plant extracts [7, 57].

#### GIC characterization

In reviewing the included studies on propolis-modified GIC, it is noteworthy that all of them overlooked the characterization of GIC itself. This lack of characterization limits the understanding of how propolis interacts with GIC and affects its overall performance in clinical applications, highlighting a significant gap in the current research landscape. The use of analytical techniques like Fourier Transform Infrared Spectroscopy (FTIR) and X-ray Diffraction (XRD) could have provided valuable insights into the structural changes and interactions occurring within the modified GIC, enhancing the overall understanding of its properties and efficacy [90–94].

### Antimicrobial effect

Propolis-modified GIC demonstrated a clear enhancement in antimicrobial activity, particularly against *S.mutans* and *L.acidophilus*. Data from the studies showed that ethanolic extracts of propolis EEP at higher concentrations, such as 25% and 50%, consistently improved antibacterial efficacy. Long-term evaluations indicated that GIC modified with propolis sustained its antimicrobial effect for up to 90 days, outperforming conventional GIC and other antimicrobial modifications like chlorhexidine. The presence of flavonoids, phenolic acids, and other bioactive compounds in propolis disrupt microbial membranes and inhibits biofilm formation, which is especially effective in reducing cariogenic bacterial populations.

#### Streptococcus*** mutants***

Although only a few studies concluded that propolis added to GIC did not confer any antibacterial activity against *S. mutans* and actually decreased the flexural and shear bond strength of the material [43, 45], the addition of EEP to GIC was primarily shown to inhibit the adhesion of *S. mutans* to the cement surface, with concentrations higher than 25% exhibiting better antimicrobial activity [37, 38, 41, 42]. The antibacterial effect against *S. mutans* was found to be concentration-dependent, with higher concentrations of EEP diminishing the participation of *S. mutans* in the formation of dental biofilm [41]. GIC modified with EEP showed improved antibacterial properties against *S. mutans*, with concentrations of 50% EEP being effective in eliminating oral microbiomes, while concentrations under 25% EEP were less effective [36].

Propolis exhibits inhibitory effects on the production of water-insoluble glucans by glucosyltransferases *S. mutans* generates glucosyltransferases and creates glucans from sucrose (especially, water-insoluble glucans), facilitating the attachment of *S. mutans* and other oral bacteria on tooth surfaces, and aiding in the development of dental plaque [15]. GIC with EEP also exhibited a statistically significant anti-adherence impact on *S. mutans*, which is particularly hydrophobic, and this hydrophobic bonding is considered an essential factor in their adherence capabilities. It is therefore suggested that the anti-adherence actions of EEP with GIC may have modified this natural hydrophobic connection between the bacteria and the smooth glass surfaces [37].

It was also proved that EEP took part in inhibiting the activity of glucosyltransferase enzymes (GTFs) thereafter preventing dental caries and diseases associated with plaque by blocking the virulence factor [38]. Authors have revealed that there was no antibacterial difference among GIC modified with propolis and those prepared chlorhexidine [50]. The antibacterial effects of GIC against. *S. mutans* were influenced by the type of GIC and the concentration of EEP. Overall, the combination of GIC and EEP reduced the Minimum Inhibitory Concentration (MIC) against *S. mutans* by two folds, indicating a doubling of the antimicrobial activity [39].

#### Lactobacillus

GIC with propolis showed significant antimicrobial activity against *Lactobacillus acidophilus* on day 7 and day 14, with a mean zone of inhibition of 11.70 mm and 9.70 mm, respectively. The antimicrobial efficacy of propolis against *Lactobacillus acidophilus* may be attributed to the synergistic effects of flavonoids present in propolis, which disrupt microbial membranes or cell walls, leading to functional and structural defects [46].

The addition of EEP to GIC resulted in superior inhibition against *Lactobacillus*, attributed to the antimicrobial properties of propolis components like galangin and caffeic acids, which inhibit bacterial growth and, suggesting that these additives can be effective in inhibiting the growth of *Lactobacillus* and preventing dental caries [43].

#### Candida*** albicans***

It was observed that the *Candida albicans*, a fungus, was less sensitive to propolis treatment than the bacteria. GIC containing propolis extract exhibited significant antifungal activity against *Candida albicans*, with a notable inhibition zone diameter, indicating the potential of propolis as an effective antifungal agent. This can be attributed to its bioactive compounds like flavonoids and phenolic acids, which have been reported to possess antifungal properties by disrupting fungal cell membranes and inhibiting fungal growth [34]. EEP has been demonstrated to exhibit antifungal properties similar to those exhibited by nystatin [43].

#### Streptococcus*** salivarius***

GIC exhibited significant antibacterial effects against *Streptococcus salivarius*, leading to a notable decrease in cellular viability after 24 h of bacterial contact. This antibacterial activity was specific to *S. salivarius*. This improvement in antimicrobial efficacy against *S. salivarius* can be attributed to the release of flavonoids from the propolis-modified GIC [34].Overall, the antimicrobial activity of propolis-modified GIC consistently outperformed unmodified GIC, with 50% concentrations demonstrating the highest efficacy across most studies. This was particularly evident against *S. mutans*, where the inhibition zones significantly increased at 50% propolis concentration compared to unmodified GIC.

For instance, *Meneses *et al*.* [41] reported that the inhibition zones tripled when GIC was modified with 50% propolis, highlighting its superior antibacterial performance. Similar results were observed against *Lactobacillus acidophilus* and *Candida albicans*, where 50% propolis consistently produced larger inhibition zones and stronger antimicrobial effects than lower concentrations. While 25% propolis concentrations already showed notable improvements in antimicrobial activity, the studies collectively confirm that 50% concentrations delivered the most substantial and sustained antibacterial and antifungal effects. Propolis is biocompatible and more effective compared to synthetic antimicrobials like chlorhexidine regarding its anti-adherence activities on *S. mutans* [37]. Unlike antibiotics, it poses no risk of resistance development, making it a safer long-term solution for dental applications [45].

### Mechanical properties

It’s highly important to investigate the mechanical strength when combining GIC with propolis, as GIC is typically brittle materials. Understanding these mechanical properties is essential for evaluating the overall performance and suitability of these materials in clinical applications [49].

#### Bond strength

The shear bond strength of restorative materials to dental substrate is the key factor in restorative dentistry because it influences the clinical longevity of the restorative material. Glass ionomer cements are brittle substances, and evaluating bond strength is crucial for assessing the longevity of these materials [39].Using the inner surface of the tooth tissue that had undergone various treatments before GIC filling, a non-standardized pull-out test was employed as a unique technique. Because it encompasses a larger dentin surface, this test offers a more accurate method of assessing GIC-tooth adhesion, even if it is not standard in all labs. It’s crucial to perform acid etching before treating the cavity with propolis, otherwise the nonpolar component of propolis will cover the internal surface of the tooth restricting the adhesion of GIC to the dentin [32].

The introduction of EEP into GIC was observed to diminish the bond strength of the mixture, which may be attributed to disruptions in the network formation inherent to GIC systems, consequently resulting in detrimental effects [39]. The shear bond strength test results indicated that the 25% and 50% propolis groups did not show significant differences in shear bond strength, but both had significantly lower shear bond strength values compared to the control group [36, 42]. On the other hand, the addition of EEP to GIC was recorded to not negatively impact the shear bond strength results, indicating that the modified GIC maintained their adhesive properties [41, 44].

#### Compressive strength

Compressive strength is a critical property for restorative materials, particularly for those used in dental applications. It indicates the material’s ability to withstand forces during mastication [47]. glass ionomers are inherently brittle, making the assessment of their compressive strength essential for evaluating their performance in clinical settings [47]. In terms of compressive strength after adding propolis in various concentrations, authors have recorded that propolis reduced compressive strength [32, 45, 47]. This decrease was attributed to components in propolis, such as flavonoids, which can form chelates and bind with available divalent (calcium) or trivalent (aluminum) ions, thereby interfering with the curing process [32]. *Azalia *et al. [46] also found that the addition of ethanolic extracts of propolis (EEP) to GIC decreased its compressive strength, with mean values of 118.06 MPa for unmodified GIC and 103.17 MPa for 25% EEP, while 30% and 35% EEP groups showed even lower strengths (79.18 MPa and 77.03 MPa, respectively). Overall, GIC modified with 25% EEP may still be a promising restorative material due to its antibacterial properties despite the reduction in strength. The decrease in compressive strength is critical because it suggests that while propolis may offer some benefits, such as antimicrobial properties, it compromises the structural integrity of the GIC. This is particularly important in dental restorations, where materials must endure significant forces without failing [47]. *De Morais *et al. [36] found that adding ethanolic extract of red propolis (EERP) did not significantly affect the compressive strength of GIC, particularly at the 25% concentration, which had the least impact on mechanical properties. The study showed that while higher concentrations of EERP (25% and 50%) demonstrated strong antimicrobial activity, they did not compromise the compressive strength of the cement.

#### Flexural strength

In the subsequent studies, flexural strength assessments were conducted to evaluate the mechanical characteristics of GIC enhanced with propolis aqueous extract. *Biria *et al*.* [35] investigated the effect of propolis aqueous extract on two types of Fuji GIC,comparing high-viscosity (Fuji IX) and low-viscosity (Fuji II) cements. In the Fuji II GIC groups, the addition of 25% and 50% propolis did not result in significant changes in flexural strength compared to the control group (*P* = *0.096*). Similarly, the Fuji IX GIC groups with 25% and 50% propolis showed no significant differences in flexural strength (*P* = *0.905*). However, the study concluded that Fuji IX GIC had significantly higher flexural strength values than Fuji II GIC (*P* = *0.001*).

The study highlighted that Fuji IX, being a high-viscosity GIC, exhibited higher flexural strength than Fuji II, a low-viscosity GIC. This difference is likely due to the inherent properties of the materials, where high-viscosity GIC are designed to provide greater strength and durability, making them more suitable for load-bearing applications in dentistry. The study reported no significant differences in flexural strength among the groups containing propolis compared to the control groups for both Fuji II and Fuji IX GIC. The authors explain that the addition of propolis aqueous extract did not negatively impact the mechanical properties of the GIC. This could be due to the nature of the propolis aqueous extract and its interaction with the GIC components, which did not compromise the structural integrity of the materials. Interestingly, *Panahanadeh *et al*.* [43] strongly disagreed with the findings of the previous study. In their research, the control group exhibited significantly higher flexural strength compared to the groups with 25% and 50% aqueous extract of Iranian propolis.

This suggests that the addition of propolis had a negative impact on the mechanical properties of the resin-modified GIC (RMGIC), with the 50% propolis group scoring the lowest flexural strength. In contrast, the control group had the highest flexural strength. Additionally, the 50% propolis group showed significantly lower flexural strength than the 25% propolis group, with a p-value of *0.006*.

#### Microhardness

*Altunsoy *et al*.* [33] highlighted the beneficial impact of adding EEP to GIC on microhardness. The results show a significant increase in Vickers’ hardness number (VHN) with higher concentrations of EEP (10%, 25%, and 50%), demonstrating a linear positive correlation between EEP concentration and hardness. The results confirm that the improvement is due to an intrinsic improvement in the material’s mechanical properties.

The enhancement of the microhardness in EEP incorporated GIC samples may be due to the interaction between GIC and EEP molecules. Numerous aromatic fatty acids and phenolic compounds exist within the EEP molecule. A chelation reaction takes place between the phenolic hydroxyl and the carboxyl group of GIC. EEP can function as a separator for dissociative carboxyl, enabling highly active poly-salt connections and cross-linking. Ethanol in EEP likely acts as a solvent, facilitating the integration of propolis into the GIC matrix. Enhanced hardness means the GIC is likely to perform better in terms of longevity, withstanding masticatory forces, and maintaining its structural integrity over time [33]. *De Morais *et al. [36] provided an important insight by comparing the microhardness of two different GIC brands, Meron and Riva. Their Vickers microhardness analysis revealed no statistically significant differences in microhardness between these two materials, suggesting that the type of GIC, whether Meron or Riva, did not significantly affect the hardness. This finding indicates that, at least for these two GIC brands, the material composition or formulation does not have a substantial impact on their microhardness, implying that both brands may offer comparable performance in terms of resistance to localized deformation in clinical settings. The study also revealed that the different concentrations of EERP (10%, 25%, and 50%) did not lead to statistically significant differences in microhardness for either the Meron or Riva GIC brands. This indicates that the addition of EERP, regardless of concentration, did not significantly affect the hardness of these GIC materials [36].

#### Diametral tensile strength

In the study by *De Morais *et al*.* [36] the diametral tensile strength (DTS) was evaluated, and it was found that the addition of 25% EERP did not significantly alter the DTS compared to conventional GIC. While the study primarily emphasized the 25% concentration, which was shown to be optimal for maintaining DTS without significant degradation, the effects of other concentrations (10% and 50%) were not the main focus but may have different impacts on tensile strength. This finding highlights that 25% EERP strikes a balance in enhancing the properties of GIC without compromising their tensile strength, making it a promising concentration for potential dental applications [36].

Reduction in the DTS seen in other studies could be attributed to potential interference with the chemical reactions of the GIC, which might cause an increase in unreacted particles within the material. The presence of these unreacted particles could weaken the overall structure of the cement, leading to compromised mechanical properties [47, 49]. *Troca *et al*.* [49] highlighted that the impact of propolis on tensile strength was material dependent. This means that different types of GIC responded differently to the incorporation of propolis, which is crucial for understanding how to optimize the use of propolis in dental materials. The results indicated that the addition of propolis to GIC had a negative effect on the DTS, particularly for the ChemFlex material.

#### Fluoride release

Released fluoride ions are key property when choosing GIC, Fluoride is efficient in inhibiting the demineralization process and promotes the remineralization process [45]. The reported results could be explained by the physical presence of EEP inside the matrix of. It also could be due to the increased solubility of GIC after adding EEP [95]. Also, It could also be because of the nature of propolis, which is obtained from plants and some of these plants do contain fluorides [45].

#### Aesthetic

The color of plant extracts is a critical yet often overlooked aspect in research studies. Variations in the color of plant extracts can arise from several factors, including the type of plant, the specific bioactive compounds present, and the extraction method employed. For example, aqueous extracts usually yield lighter, more transparent colors, ranging from pale yellow to green, due to water-soluble compounds like flavonoids and tannins [74, 75], However, *Panahandeh et. al* [3]*.* recorded their obtained aqueous extract had a dark brown color. In contrast, ethanolic extracts often produce deeper, more vibrant colors, such as amber, brown, or dark green, because ethanol is more effective at extracting a wider range of pigments, including chlorophylls and carotenoids [76, 77]. The color of the extract can provide insights into its composition and concentration, which may affect the aesthetics of the final GIC product.

For instance, the incorporation of Ethanol Extract of Pomegranate (EEP) into GIC resulted in a yellowish tint, attributed to the natural yellow–brown color of the EEP. When mixed with a light-colored GIC liner, this led to a darker final material. Discoloration has been noted as a challenge in other studies involving propolis modified GIC [44]. However, this issue is less critical when the extract is used as a liner since liners are typically covered by other restorative materials [30]. Understanding these color variations is crucial for achieving the desired visual and material properties of GIC in dental applications, as overlooking the color of PE might lead to challenges in meeting aesthetic outcomes [44]. Additional research concerning the separation of the active component must be conducted, along with the removal of the extract’s color effect on GIC, which is currently unacceptable [78].

#### Handling properties

Higher concentrations of propolis (50%) caused yellow discoloration in GIC, which, while functional for posterior restorations, limits its application in anterior teeth where aesthetics are critical. Low to moderate concentrations (10–25%) had a minimal impact on handling properties such as setting time and viscosity. propolis-modified GIC exhibited increased water sorption compared to the pure material, which suggests changes in the consistency and possibly the handling properties of the cement. Specifically, the lyophilized form of propolis led to greater water sorption than the ethanolic extract, indicating that the form of the extract can significantly impact the consistency of the modified GIC [49].

However, it is important to note that no study measuring the color stability of propolis-modified GIC has been reported, which leaves a gap in understanding its long-term aesthetic performance. This lack of data highlights the need for further research to evaluate the material’s color stability over time, especially in clinical practices where restorations are exposed to staining agents.

## Challenges and future directions

The use of propolis in glass ionomer cement (GIC) presents several challenges and future opportunities.

- It is important to acknowledge that this systematic review primarily includes in vitro studies, which, while valuable for establishing baseline properties, do not fully replicate the complex conditions of the oral environment. Factors such as saliva, bacterial biofilm formation, temperature fluctuations, and pH variations influence the clinical performance of dental materials. Future research should incorporate in situ and clinical studies to evaluate the long-term antimicrobial effectiveness and mechanical stability of propolis-modified GIC under oral conditions.
- One of the critical limitations identified in this review is the lack of detailed chemical characterization of both the propolis extract and the modified GIC. Most studies did not perform FTIR, XRD, or other advanced analyses to identify the active bioactive compounds within the propolis extract, such as flavonoids, phenolic acids, and aromatic esters. Without this characterization, it remains unclear which specific components contribute to the observed antimicrobial and mechanical effects. Future studies should prioritize comprehensive chemical characterization to establish a direct correlation between the composition of propolis and its impact on GIC properties. Such analyses would also enable optimization of propolis concentrations for enhanced clinical performance.
- A notable concern associated with propolis incorporation into GIC is its potential to cause yellowish discoloration, particularly at higher concentrations (≥ 25%). This discoloration could limit its application in anterior restorations where aesthetics are a priority. However, none of the reviewed studies assessed the long-term color stability of propolis-modified GIC when exposed to factors such as staining beverages, aging, and light exposure. Future research should investigate the color stability and possible modifications to mitigate discoloration, such as refining propolis extraction methods or incorporating additional stabilizers to maintain aesthetic acceptability.
- Variability in extraction methods, GIC brands (e.g., Meron, Fuji IX, Ketac Cem), and propolis concentrations has significantly influenced results, highlighting the need for standardized protocols for propolis preparation. Mechanically, propolis concentrations ≥ 50% tend to reduce compressive and bond strength, limiting the material’s structural durability. Future directions include the development of standardized protocols for propolis extraction and preparation to minimize variability and ensure consistency in outcomes.
- Exploring hybrid modifications, such as combining propolis with other bioactive agents, may help address its mechanical limitations while maintaining its antimicrobial properties. Green synthesis methods that integrate propolis with nanoparticles hold promise for enhancing its potential applications. Lastly, assessing the pulpal response of propolis-modified GIC is critical, especially for deep carious lesions, and should be prioritised in future research.

## Conclusion

Propolis-modified GIC represents a promising advancement in restorative dentistry, offering enhanced antimicrobial activity, particularly against Streptococcus mutans, Lactobacillus, and Candida albicans, and, in some formulations, improved physical or mechanical properties. Concentrations around 25% often provided a balance between antimicrobial efficacy and acceptable mechanical performance, whereas higher concentrations (e.g., 50%) further improved hardness but could reduce compressive and bond strength.

However, the available evidence is limited by considerable heterogeneity in study design, including differences in GIC brands, propolis botanical origins, extraction techniques, and incorporation methods. The absence of standardized chemical characterization of propolis extracts (e.g., FTIR, GC–MS, HPLC) remains a critical gap, making it difficult to determine which specific bioactive compounds are responsible for the observed effects and to ensure reproducibility across studies. Methodological weaknesses, such as lack of randomization, blinding, and sample size calculation, further reduce the generalizability of current findings.

From a clinical perspective, while the antibacterial potential of propolis-modified GIC is encouraging, long-term aesthetic properties such as color stability, translucency, and gloss retention remain unexplored. This is especially important for anterior restorations where esthetic integration is critical.

## Data Availability

The data used to support the findings of this study are included within the article.
